# Mitigating the effects of granular scattering using cepstrum analysis in terahertz time-domain spectral imaging

**DOI:** 10.1371/journal.pone.0216952

**Published:** 2019-05-16

**Authors:** Omar B. Osman, M. Hassan Arbab

**Affiliations:** Department of Biomedical Engineering, Stony Brook University, Stony Brook, New York, United States of America; Oregon State University, UNITED STATES

## Abstract

Terahertz (THz) imaging is a widely used technique in the study and detection of many chemicals and biomolecules in polycrystalline form because the spectral absorption signatures of these target materials often lie in the THz frequencies. When the size of dielectric grain boundaries are comparable to the THz wavelengths, spectral features can be obscured due to electromagnetic scattering. In this study, we first investigate this granular scattering effect in identification of chemicals with THz spectral absorption features. We then will propose a signal processing technique in the so-called “quefrency” domain to improve the ability to resolve these spectral features in the diffuse scattered THz images. We created a pellet with *α*-lactose monohydrate and riboflavin, two biologically significant materials with well-known vibrational spectral resonances, and buried the pellet in a highly scattering medium. THz transmission measurements were taken at all angles covering the half focal plane. We show that, while spectral features of lactose and riboflavin cannot be distinguished in the scattered image, application of cepstrum filtering can mitigate these scattering effects. By employing our quefrency-domain signal processing technique, we were able to unambiguously detect the dielectric resonance of lactose in the diffused scattering geometries. Finally we will discuss the limitation of the new proposed technique in spectral identification of chemicals.

## Introduction

Two-Dimensional spectroscopic chemical mapping of THz [1–3] and infrared (IR) [4] images are valuable tools in biomedical, pharmaceutical and security applications. This utility is often because the high specificity in the signal contrast of the images is due to the existence of unique dielectric resonance modes in the absorption spectra of the molecules. In particular, characteristic spectral features of many biomolecules [5, 6], pharmaceuticals [7], illicit drugs [8], and explosive materials [9–11] reside in the THz regime. While some of these spectral features, like the torsional vibrational modes of tryptophan [12, 13], are due to intramolecular vibrational modes, THz radiation can also induce intermolecular interactions such as in polycrystalline saccharides [14]. This means that the source of THz spectral absorption features can be structural, molecular, or a combination of the two [8, 15].

Granular scattering in THz time-domain spectroscopy (THz-TDS) measurements can diminish image quality and obscure spectral features [16, 17]. Scattering can be especially detrimental when measuring illicit chemicals and explosives because target materials typically contain dielectric heterogeneities and geometric features in the form of plastic fillers, air voids, and granularity [16]. When the sizes of these heterogeneities are on the same order of the THz wavelengths, they give rise to significant electromagnetic scattering.

Experimentally, electromagnetic scattering can be observed as a loss of energy from the incident signal and the redistribution of energy that propagates in other directions [18]. The mechanisms behind the rough surface and granular scattering are different. For example, samples with rough surfaces can be modeled by the Kirchhoff Approximation if the surface height variations have a Gaussian distribution [18–20]. For granular materials, on the other hand, the extent of the redistribution of energy depends on the shape, concentration, dielectric contrast and orientation of the objects that are causing the scattering [16–18]. These parameters play a large part in how the mechanisms of granular scattering are modeled. For example, densely packed media has been modeled using a quasi-crystalline approximation [16] and radiative transfer theory [21], whereas the energy loss from loosely packed media has been modeled by a simple power law [17].

Previous studies have used a wide range of laboratory and signal processing techniques to mitigate the effect of electromagnetic scattering from both granular materials and rough surfaces. Shen *et al*. suggest that finely milling the material as well as increasing the sample’s packing density will reduce the effect of granular scattering [17]. However, physically manipulating the sample (e.g. pressing it into a pellet) may not be practical outside of a laboratory setting. Other studies have shown that averaging spatial measurements [17] and signal processing techniques [19, 22–25] can remove scattering effects computationally. Wavelet-based techniques have been shown to be a useful tool for identifying spectral features that are obscured by rough-surface scattering effects [22, 23, 26]. More recently, spectral dynamics analysis and integral correlation criteria have been shown to be effective methods for detecting materials without spatial averaging [27].

Schecklman *et al*. has shown that filtering and analysis of THz data in the quefrency domain can retrieve obscured spectral features from samples with rough surfaces in a reflection geometry [19]. Additionally, this study improved signal to noise by taking the average of the THz spectra from 30 spatial locations on the sample and averaging the specular and all diffuse scattered angles [19]. The terms “cepstrum” and “quefrency” are anagrams of “spectrum” and “frequency”. To obtain the complex cepstrum of a signal in the quefrency domain, the Fourier transform of the original signal in the frequency domain is computed. Signal processing techniques in the quefrency domain have been used in several applications—speech processing, geophysics and medical imaging, to name a few [28].

In this study, we created a sample with a pellet made from two materials with known THz spectral features, at 0.53 THz for *α*-lactose monohydrate and 1.02 THz for riboflavin, and buried it in a non-absorbing and highly-scattering polyethylene particle medium. The sample was raster-scanned, and the scattered THz signal was gathered by using a rotating arm to capture all angles in the transmission focal plane. First, we will investigate the effect of granular scattering at various diffuse geometrical measurement angles. We will show that cepstrum filtering can resolve the spectral features and provide a method for achieving chemical mapping using only the incoherent diffuse scattered THz measurements. Finally, we will discuss bandwidth limitations of the new quefrency-domain analysis technique.

## Materials and methods

### Sample preparation

**Fig 1:**
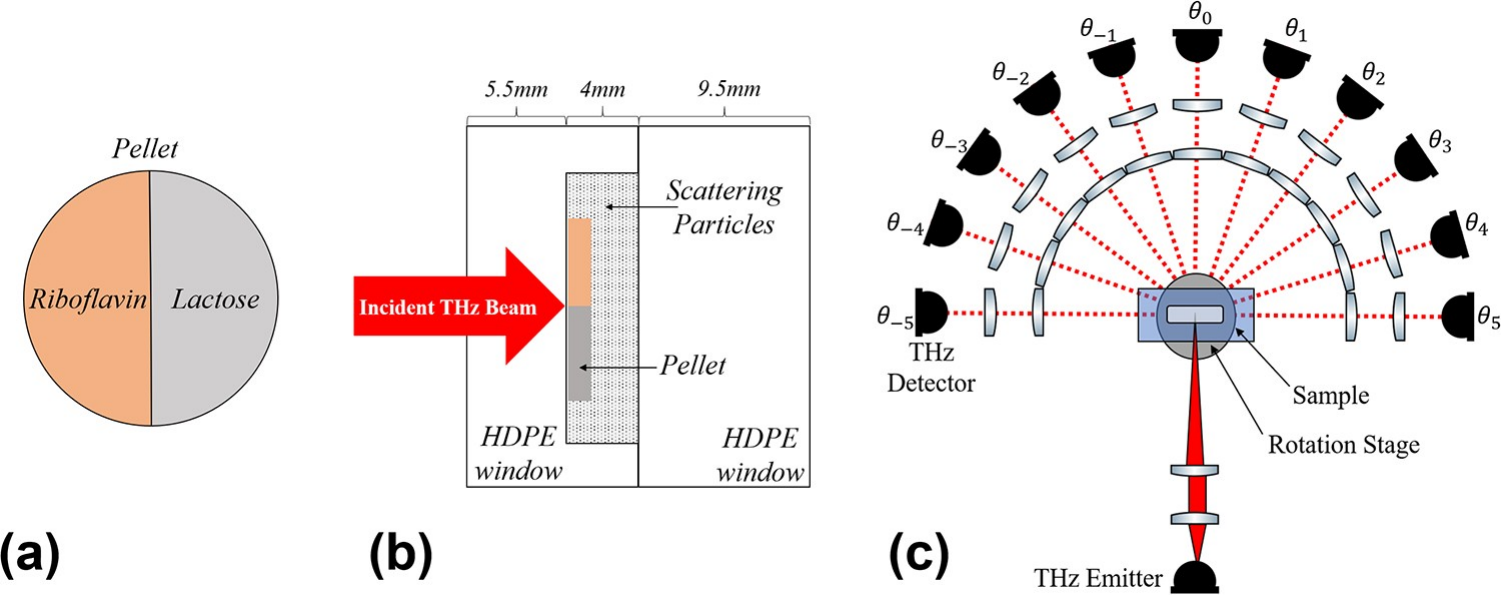
Sample and experimental setup. (a) The pellet with two different materials with unique spectral peaks in the THz regime is shown. (b) The sample location is shown in a cross-section of the sample holder which is made from two HDPE slabs. (c) The optical setup used for measuring diffused granular scattering signal is depicted. The THz detector arm was on a rotation stage and measurement angles, *θ*_±n_, were in steps of 18°.

The imaging target materials were *α*-lactose monohydrate and riboflavin (Sigma-Aldrich, Inc). These materials were selected because of their biological significance and their unique spectral features in the THz range [7]. Lactose is a disaccharide that can be found in mammalian milk [29]. Riboflavin, or vitamin B2, is an essential nutrient that is a precursor to important metabolic coenzymes [30]. The spectral absorption feature of lactose at 0.53 THz is associated with a hindered rotational mode in the B-axis of the crystal and is a relatively sharp and easily identifiable peak [31]. On the other hand, riboflavin has a much broader absorption peak feature around 1.02 THz which has not been as rigorously studied. Each material was combined with high-density polyethylene (HDPE) powder (Micropowders, Inc.) with a mean particle size of 5-7 *μ*m as a binding agent for mechanical strength of the pellet. The powder mixture was 70% HDPE and 30% target material by weight. The mixtures were pressed into 50 mm diameter pellet under 3000 psi for 30 minutes. Each material mixture was made into a separate pellet and a razor blade was used to cut the pellet into a semicircle shape, as shown in Fig 1a. Under these conditions, 3 g of the total mixture material compressed into a 2 mm pellet.

The sample holder, depicted in Fig 1b, was made from two slabs of 9.5 mm thick HDPE slabs with a square reservoir machined into one of the slabs (75 mm length, 75 mm width, and 4 mm depth). We placed each semicircle pellet in the center of the square and filled the remaining volume with approximately 10 g of HDPE particles (Micropowders Inc.) with a mean particle size of 180 *μ*m for an effective packing density of 0.59 g/cm^3^. The packing density and particle size were selected based on scattering loss experiments done in [17], suggesting these parameters would cause sufficient scattering to obscure the spectral features in our sample.

### THz setup

Scattering measurements were taken with an ASynchronous OPtical Sampling (ASOPS) THz-TDS system (Menlo Systems, Inc). This setup uses two 1550 nm femtosecond fiber lasers at slightly offset repetition rates. The ASOPS system was set to a repetition rate of 100 MHz and a difference frequency of 100 Hz. One laser pumped an InGaAs/InAlAs photoconductive antenna (PCA) emitter and the other laser was coupled to a LT InGaAs/InAlAs PCA receiver. Moreover, the ASOPS system has a dynamic range of approximately 80 dB. To acquire scattered THz signal, we built a transmission configuration where the THz detection arm was mounted on a rotation stage, shown in Fig 1c. The emitted THz beam was first collimated with a plano-convex lens (TPX 50 mm EFL, Menlo Systems, Inc) and then focused onto the sample using a plano-convex lens with a longer focal length (PTFE 100 mm EFL lens, Thorlabs, Inc). The detection arm consisted of an identical plano-convex PTFE lens to recollimate the beam after propagation through, and scattering by the sample. An identical plano-convex TPX lens was used to focus the beam on the detector. The detection arm was built on an optical rail which was mounted on a 360° rotation stage. Using knife-edge measurements, the collimated beam diameter was determined to be approximately 15 mm and the focal spot size was approximately 4.3 mm. The sample was raster scanned at the focal point with a pixel size of 4 mm.

For each pixel, the THz field at the forward-scattering angle, *θ*_0_, was measured and higher-angle scattering was measured by placing the detection arm at *θ*_±n_ positions in Fig 1c. At each angle, 1000 time averages were taken before the detection arm would be moved to the next *θ*_±n_ position. The step size of *θ*_±n_ (18°) was determined by calculating the divergence angle based on the diameter of the collimated beam and spot size of the THz beam and ensuring a minimum step size to capture all THz radiation transmitted or scattered passed the focal plane.

### Signal processing

Each time-domain measurement, as a function of spatial position on the sample and the angle of the detector arm, was Fourier transformed and deconvolved, using a reference THz measurement through air,

$$\tilde{t}\left(x,y,f,\theta \right)=\frac{{\tilde{E}}_{\text{sample}}\left(x,y,f,\theta \right)}{{\tilde{E}}_{\text{reference}}\left(f\right)}.$$ (1)

The path length of the beam will be affected by granular scattering of the HDPE particles. This will result in incoherent phase shifts, so we conduct the rest of the analysis using the power transmission coefficient, *P*(*x*, *y*, *f*, *θ*), calculated by

$$\begin{array}{c} P\left(x,y,f,\theta \right)=|\tilde{t}\left(x,y,f,\theta \right){|}^{2}. \end{array}$$ (2)

The negative derivative of the deconvolved THz power with respect to frequency can show the location of the spectral features near the peak frequencies in the absorption coefficient [2]. We then compute and average the derivative across measurement angles to calculate the diffuse scattered THz signal by [19],

$$\begin{array}{c} D_{\text{avg}}\left(x,y,f\right)=\frac{1}{N_{\text{a}}}\sum_{n=\pm 1}^{N_{\text{a}}}-\frac{\partial P\left(x,y,f,\theta_{\pm \text{n}}\right)}{\partial f}, \end{array}$$ (3)

where *N*_a_ is the number of angles averaged and *θ*_±n_ is the measurement angle denoted in Fig 1. The derivative spectrum, *D*_avg_ (*x*, *y*, *f*), was windowed between 0.2 THz and 1.2 THz and a 50-point Blackman window function was added for a slow roll off to zero. The spectral derivative from diffuse scattered angles was then z-score normalized by,

$$\begin{array}{c} {\bar{D}}_{\text{avg}}\left(x,y,f\right)=\frac{D_{\text{avg}}\left(x,y,f\right)-\mu_{\text{D}\left(\text{x},\text{y},\text{f}\right)}}{\sigma_{\text{D}\left(\text{x},\text{y},\text{f}\right)}}, \end{array}$$ (4)

where *μ*_*D*(*x*,*y*,*f*)_ and *σ*_*D*(*x*,*y*,*f*)_ are the mean and standard deviation of the derivative, respectively. The cepstrum was defined by taking the Fourier transform of the normalized derivative spectrum, given by,

$$\begin{array}{c} \tilde{C}\left(x,y,q\right)=F\left\{{\bar{D}}_{\text{avg}}\left(x,y,f\right)\right\}, \end{array}$$ (5)

where *q* is the “quefrency” in picoseconds (ps) and $\tilde{C}\left(q\right)$ is the complex cepstrum. Once the THz signal is in the “quefrency” domain, we applied an appropriate Gaussian filter, *G*(*q*), defined by,

$$\begin{array}{c} G\left(q\right)=e^{\frac{{\left(q-\mu \right)}^{2}}{2\sigma^{2}}}, \end{array}$$ (6)

where *μ* and *σ* are the mean and standard deviation of the filter, respectively. The ideal values for *μ* and *σ* were calculated based on the cepstrum of each material’s extinction coefficient, *κ*, and $\tilde{C}\left(q\right)$ through HDPE scattering particles. The complex indices of refraction of the target materials, lactose and riboflavin, were determined *a priori* in a separate set of THz-TDS measurements, transmitting only through the sample pellet and without the sample holder described in Fig 1. The material parameter extraction algorithm used a gradient descent optimization to fit a model transfer function and a quasi-space optimization technique to determine the precise sample thickness [32–35]. The Gaussian filter was used in the quefrency-domain to remove the scattering effects. The filtered $\tilde{C}\left(q\right)$ signal was then returned to the frequency domain by applying an inverse Fourier transform, given by

$$\begin{array}{c} D_{\text{filtered}}\left(x,y,f\right)=F^{-1}\{\tilde{C}\left(q\right)\cdot G\left(q\right)\}. \end{array}$$ (7)

**Fig 2:**
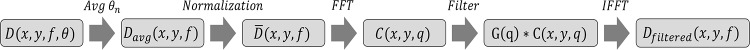
Signal processing flowchart. The flowchart describes the signal processing steps taken during the cepstrum analysis.

An overview of the signal processing steps are presented in a flowchart, shown in Fig 2.

We define a parameter called Normalized Spectral Signal, *NSS*, by taking an integral within a 0.03 THz window centered at the location of the absorption feature of each material in the derivative spectrum and dividing by the standard deviation,

$$\begin{array}{c} NSS_{\text{lactose}}=\frac{\int_{0.50THz}^{0.53THz}\;D\left(x,y,f,\theta \right)df}{\sigma_{\text{D}(\text{x},\text{y},\text{f},\theta )}}, \end{array}$$ (8)

and

$$\begin{array}{c} NSS_{\text{riboflavin}}=\frac{\int_{0.97THz}^{1.00THz}\;D\left(x,y,f,\theta \right)df}{\sigma_{\text{D}(\text{x},\text{y},\text{f},\theta )}}. \end{array}$$ (9)

In the following sections, we show the utility and limitations of the cepstrum filtering technique by plotting the *NSS* of lactose and riboflavin before and after the above signal processing steps.

## Results

### Sample characterization

**Fig 3:**
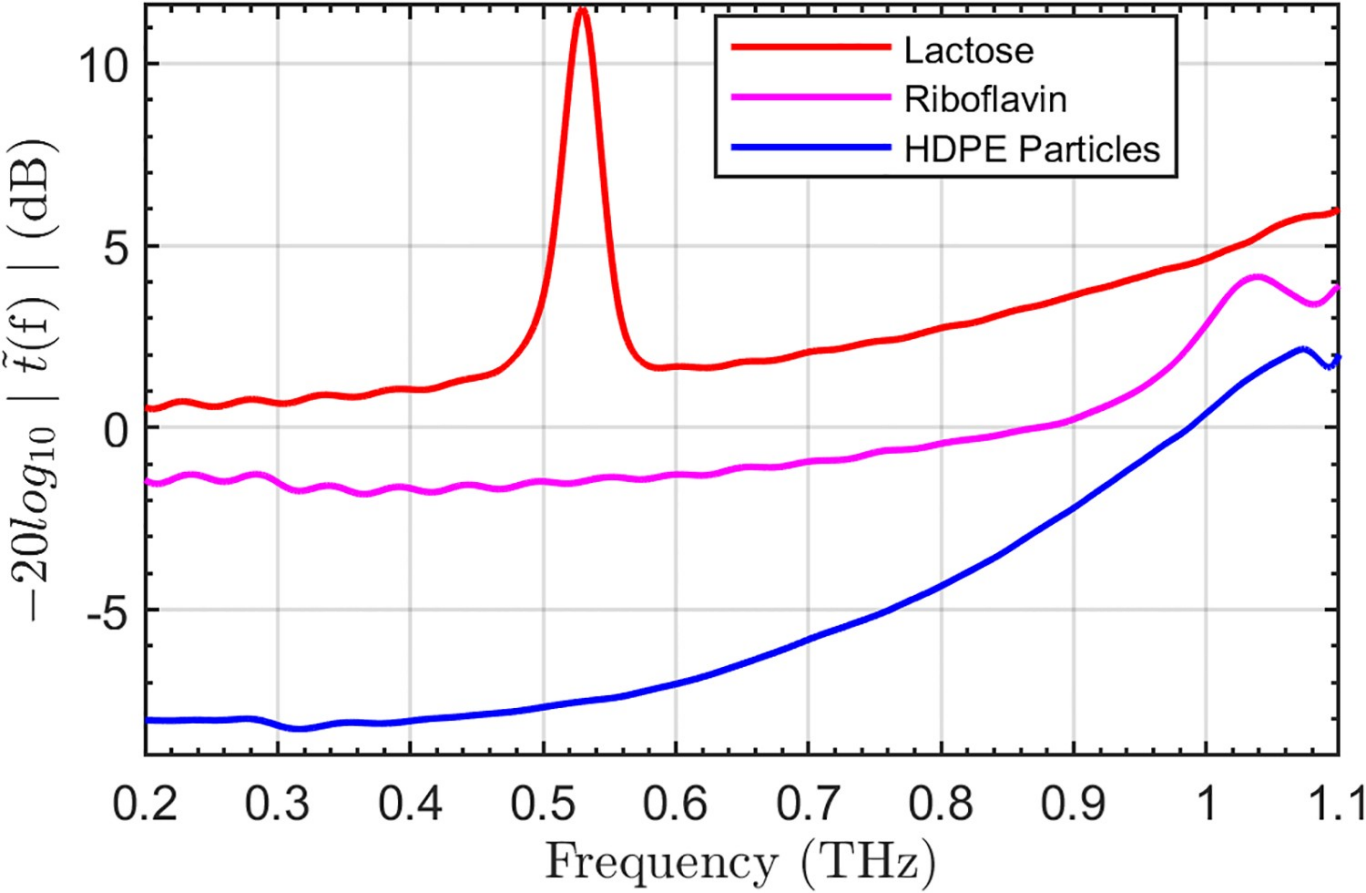
Transmission spectra. The power spectra of the 180 *μ*m HDPE particles (blue), lactose (red), and riboflavin (magenta) are shown, vertically offset for clarity.

Fig 3 shows the transmission spectra of lactose, riboflavin and HDPE scatteirng particles. Lactose has a well-known sharp absorption peak around 0.53 THz and Riboflavin shows a less distinct spectral feature around 1.02 THz. Both features are visible in the logarithmic power extinction spectra in Fig 3.

In the THz transmission spectra of scattering material, we expect to see a significant drop in coherent signal amplitude as the wavelength decreases. This phenomenon is dependent on several factors, such as the dielectric contrast [16], particle size [36] and packing density [17]. By using a packing density near that used by Shen *et al*. [17], we were able to observe a similar extinction in our power spectrum of the scattering particles, shown in Fig 3. Furthermore, because the logarithmic power extinction coefficient spectrum of the scattering HDPE particles increases with the third power as a function of frequency, we expect the riboflavin feature around 1.02 THz to be obscured more strongly than the feature of lactose around 0.53 THz. Therefore, identification of riboflavin in a scattered image is far more challenging than lactose.

### Cepstrum filtering

**Fig 4:**
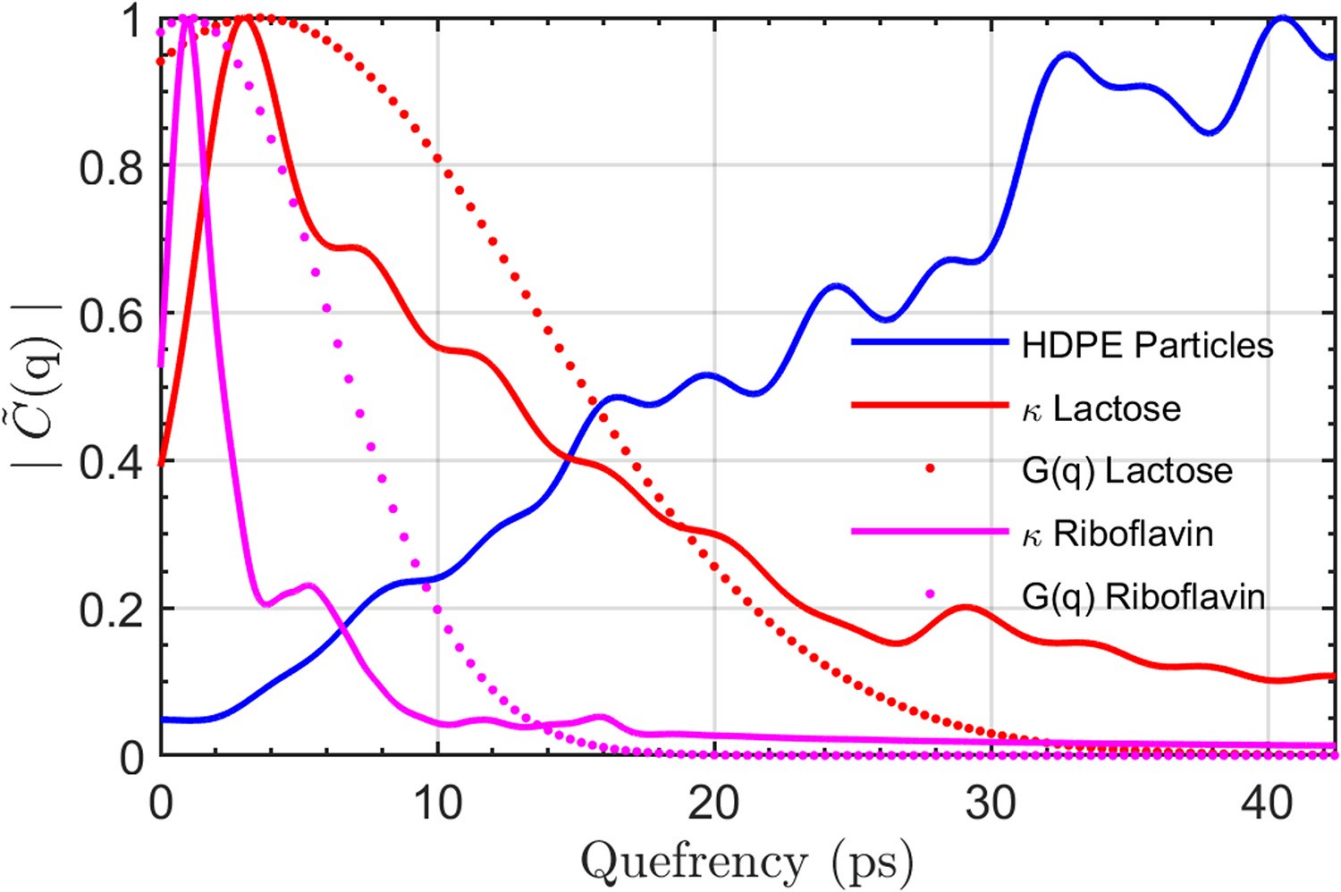
Quefrency domain analysis. The cepstrum, $\left|\tilde{C}\left(q\right)\right|$, of the 180 μm HDPE scattering particles (blue), *κ* of riboflavin (magenta) and *κ* of lactose (red), and the shape of the Gaussian filters, G(q), for each respective material are shown.

The data for the imaginary part of the index of refraction of the samples used in the calculations shown in Fig 4 were obtained in separate THz-TDS transmission measurements without the sample holder for creating the scattering effects. Fig 4 shows the cepstrum for lactose, riboflavin and HDPE scattering particles. In the quefrency domain, the extinction coefficient, *κ*, of lactose and riboflavin contain most of their information in the lower quefrency regime. Conversely, the derivative cepstrum of the scattering particles increase dramatically with quefrency between 2 ps and 40 ps. This behavior of the cepstrum suggests that a Gaussian bandpass filter with a mean of 3.5 ps and a standard deviation of 10 ps would suppress the effect of the scattering HDPE particles while retaining the signal from the lactose pixel. The cepstrum of the riboflavin *κ*, showed an even stronger bias towards the lower quefrency range and this suggests a more aggressive Gaussian bandpass filter with a mean of 1 ps and a standard deviation of 5 ps to reduce the effect of the scattering HDPE particles.

**Fig 5:**
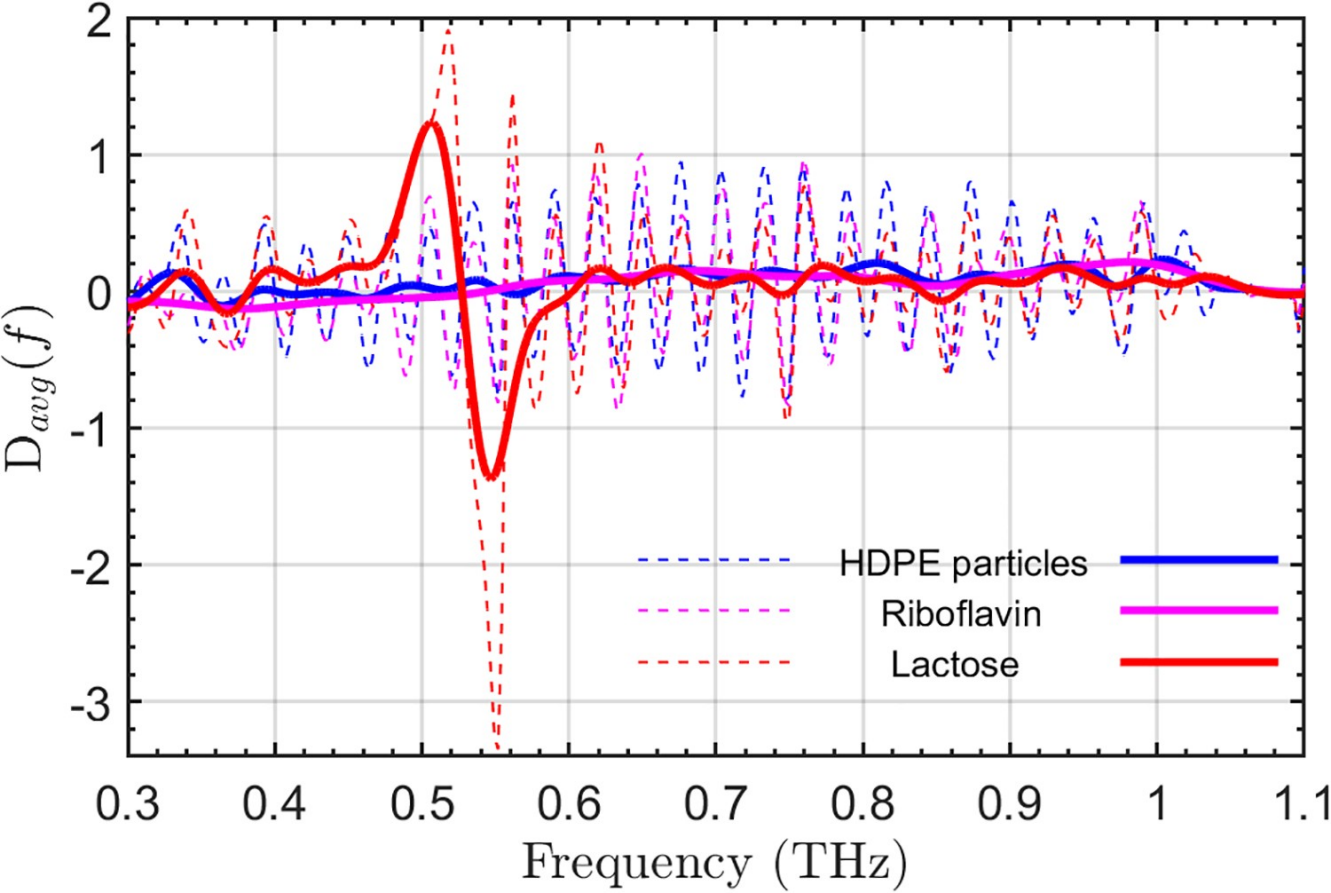
Before and after cepstrum filtering. *D*_avg_ (*f*, *θ*_0-±5_) of a single pixel propagating through only HDPE particles (blue), the lactose pellet and scattering particles (red), and the riboflavin pellet and scattering particles (magenta). The *D*_avg_ (*f*, *θ*_0-±5_) is shown before (dashed) and after (solid) cepstrum filtering.

Fig 5 shows *D*_avg_ (*f*, *θ*_0 to ±5_) of three separate pixels containing only scattering particles (blue), lactose (red), and riboflavin (magenta). After the application of the Gaussian filter in the quefrency domain, the spectral feature of lactose was more easily resolved. All THz signals are suppressed to some extent after cepstrum filtering, however the ability to differentiate the spectral feature of lactose is significantly improved while riboflavin feature (0.95 THz-1.05 THz) is barely noticeable.

**Fig 6:**
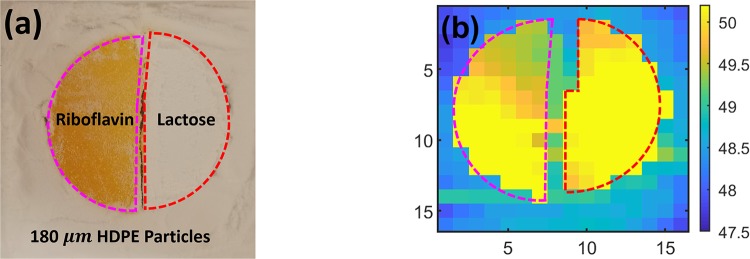
Visual and time-of-arrival image. (a) The visual image of the pellet that was buried beneath 2 mm of 180 *μ*m HDPE particles is shown with dashed outlines for lactose (red) and riboflavin (magenta). (b) The time-of-arrival image of the pellet at *θ*_0_ is also shown with outlines for each pellet material.

**Fig 7:**
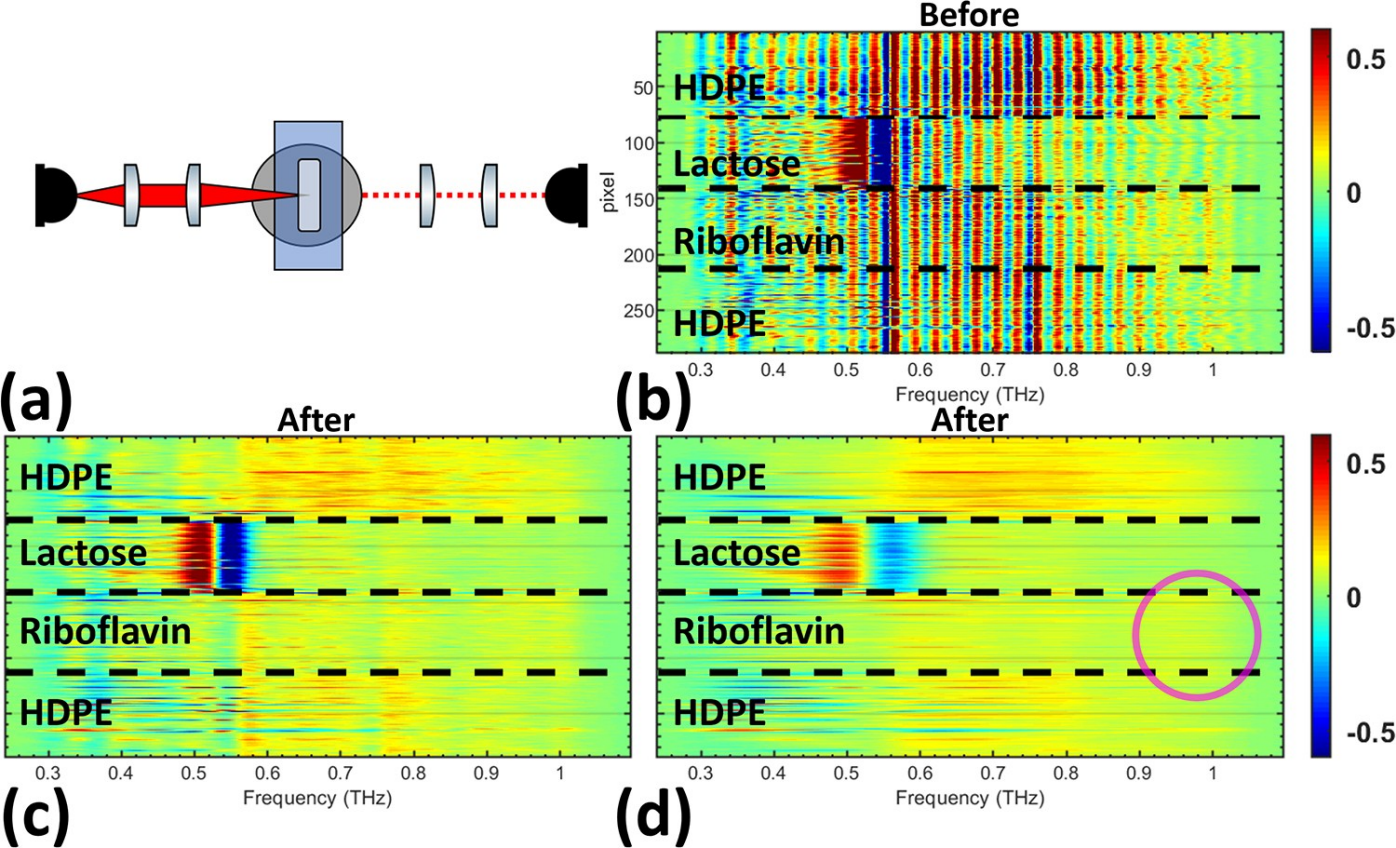
Measurements at *θ*_0_. (a) The *θ*_0_ optical configuration used in Fig 7 is shown. The derivative spectra of all pixels is shown (b) before filtering and after using the (c) lactose Gaussian bandpass filter and the (d) riboflavin Gaussian bandpass filter in the quefrency domain.

**Fig 8:**
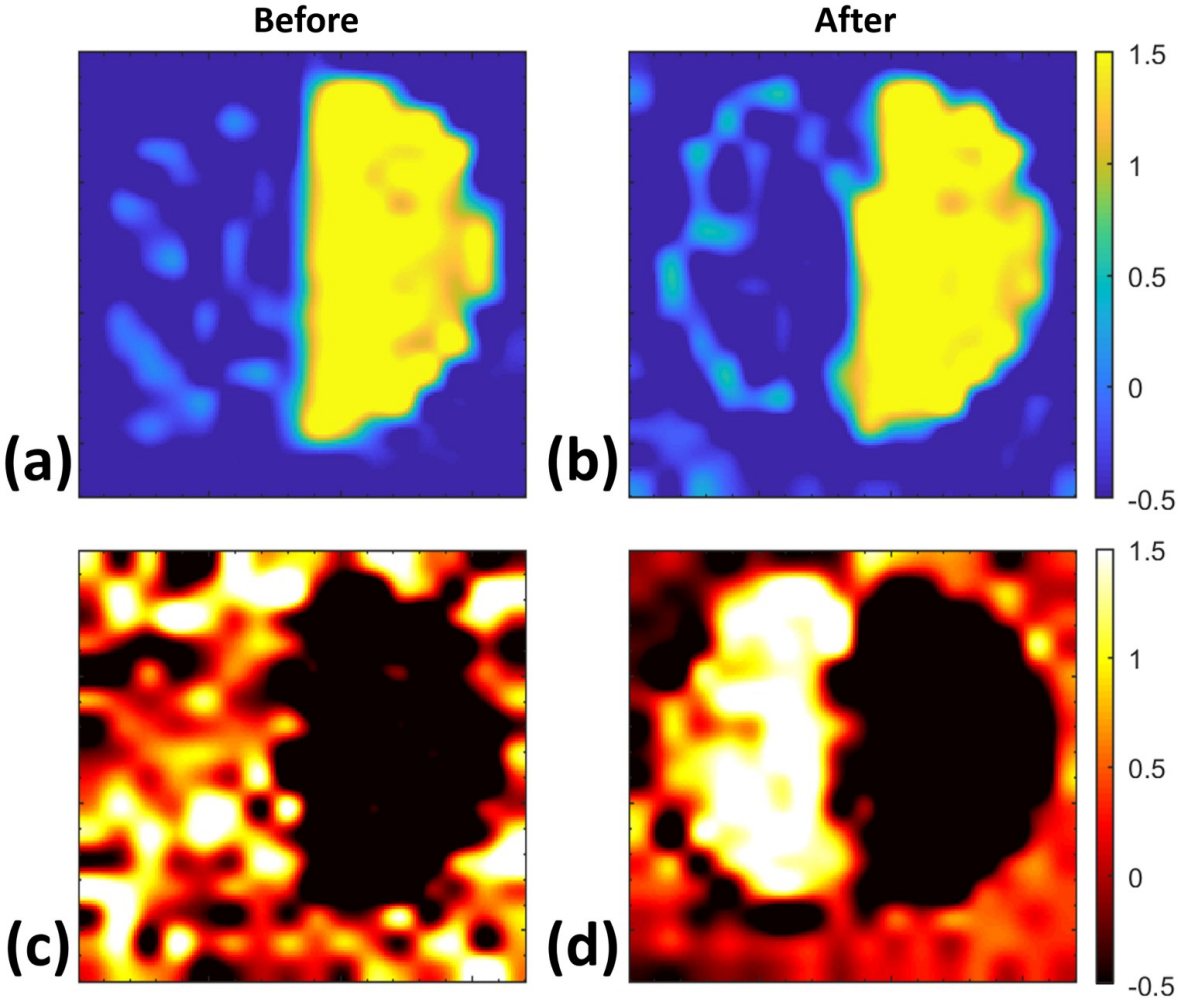
Imaging at *θ*_0_. The *NSS*_lactose_ parameter is shown (a) before and (b) after cepstrum filtering. The *NSS*_riboflavin_ parameter is shown (c) before and (d) after cepstrum filtering.

Fig 6 shows the visual and time of arrival (TOA) images obtained at *θ*_0_, which were used as our ground truth for filter improvement. Fig 6 shows that the pellet geometry can be resolved using TOA analysis. However, to discriminate between the two target materials based on their spectral features, the signal processing steps described in the previous sections were conducted. The entire derivative spectrum for each pixel before and after filtering is shown in Fig 7b–7d. The reduction in the scattering effect from the HDPE particles was clear in the analysis of the pixels. Derivative spectra shown in Fig 7 do not include the z-score normalization step in Fig 2 because signal to noise was much higher at the forward-scattered angle, *θ*_0_, than the derivative spectra from diffuse angles, which will be presented in the next section. The lactose images, Fig 8a and 8b, were created utilizing the spectral parameter from Eq 8 before and after cepstrum filtering. Although lactose pixels can readily be recognized in Fig 8a, the shape of the pellet is better resolved after cepstrum filtering in Fig 8b to resemble the ground truth images in Fig 6b. The riboflavin images, Fig 8c and 8d, were created utilizing the spectral parameter from Eq 9 before and after cepstrum filtering. In the original riboflavin image, Fig 8c, the riboflavin pellet cannot be recognized. After cepstrum filtering, however, the ability to determine the location of the riboflavin pellet in Fig 8c is significantly improved.

### Imaging using scattered power

Using a normal incidence and forward-scattered (*θ*_0_) THz measurement showed clear utility for improving the derivative spectra of riboflavin whose spectral features have been obscured by granular scattering. In biomedical and standoff detection applications, placement of the detector in transmission may not be feasible due to material absorption or other practical considerations. Furthermore, the THz detector in a reflection geometry may require the sample to be far away from the detection scheme and the sample may not have the luxury of sending the THz beam directly along the optical path [37]. Diffuse scattered measurements suffer from lower signal to noise because of the reduced power, so retrieving spectral features can be more challenging.

By using the cepstrum filtering technique, described above, on diffuse transmission measurements, we were able to reduce the scattering effects and resolve spectroscopic features of the materials. The following figures will show the effectiveness of cepstrum filtering by excluding the normal specular transmitted angle and only using measurements obtained at diffuse angles. The scattered THz energy in higher angles is reduced by 100-fold from *θ*_0_ to *θ*_±1_ and continues to drop as the angle moves farther from the forward-scattering direction.

**Fig 9:**
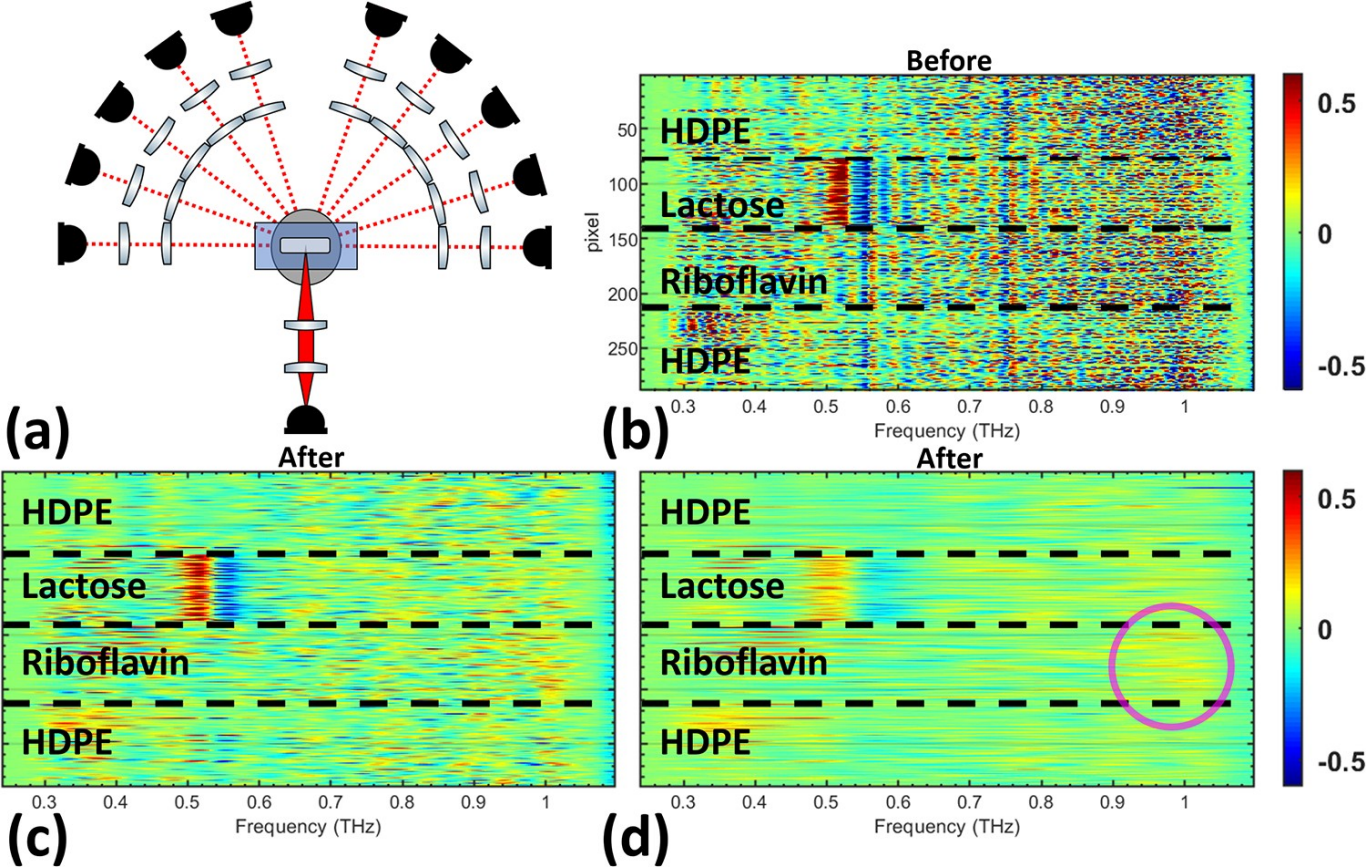
Diffuse angle measurements from *θ*_(±1 to ±5)_. (a) The optical configuration used in Fig 9 (*θ*_(±1 to ±5)_) is shown. The derivative spectra of the pixels are shown (b) before filtering and after the signal has been filtered using the (c) lactose Gaussian bandpass filter and the (d) riboflavin Gaussian bandpass filter.

In the *θ*_(±1 to ±5)_ optical configuration, shown in Fig 9, the lactose spectral feature is relatively clear before filtering. The largest change in the pixels comes from the reduction in high amplitude scattering effects across the derivative spectra for all pixels. Improvements of the riboflavin feature in Fig 9 is not as obvious as the lactose feature, but the frequency band around 1 THz has a slightly stronger signal compared to other frequencies in the spectra, as highlighted in the image with a magenta circle.

**Fig 10:**
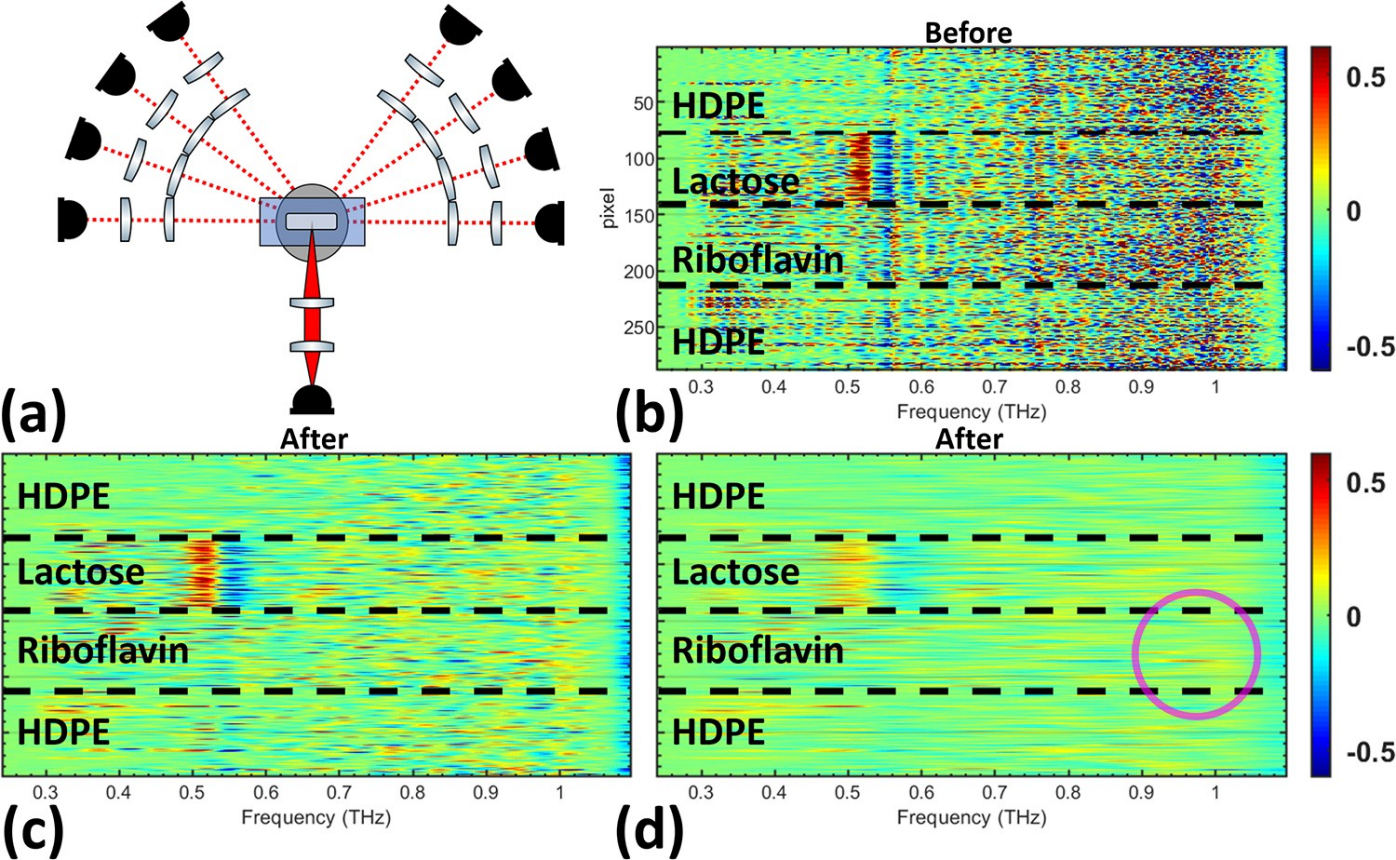
Diffuse angle measurements from *θ*_(±2 to ±5)_. (a) The optical configuration used in Fig 10 (*θ*_(±2 to ±5)_) is shown. The derivative spectra of the pixels are shown (b) before filtering and after the signal has been filtered using the (c) lactose Gaussian bandpass filter and the (d) riboflavin Gaussian bandpass filter.

**Fig 11:**
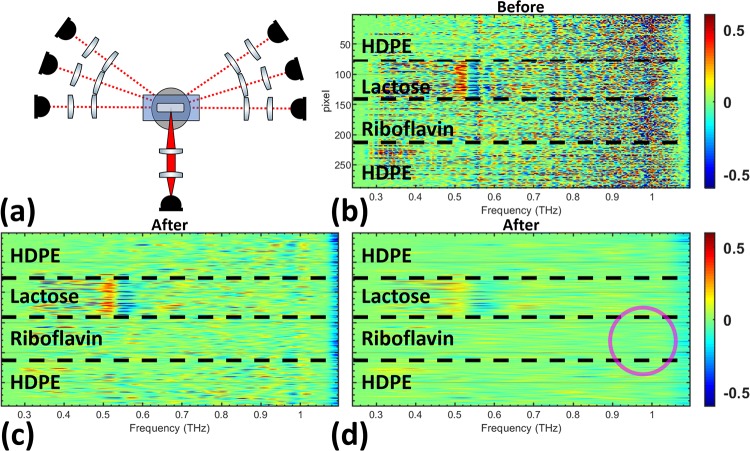
Diffuse angle measurements from *θ*_(±3 to ±5)_. (a) The optical configuration used in Fig 11 (*θ*_(±3 to ±5)_) is shown. The derivative spectra of the pixels are shown (b) before filtering and after the signal has been filtered using the (c) lactose Gaussian bandpass filter and (d) riboflavin Gaussian bandpass filter.

Similar to Fig 9, the noisy signal in all pixels across the entire derivative spectra is reduced in Fig 10. The riboflavin feature is completely obscured because of the relative weakness of its spectral feature and the strong reduction in signal to noise at *θ*_(±2 to ±5)_. Furthermore, the lactose spectral feature in Fig 10 is not as clear because of the reduced spectral energy when the detector arm is farther from the forward-scattered angle, *θ*_0_. The scattered signal also conceals the lactose spectral feature more strongly and cepstrum filtering helps to reveal it. The pixels in Fig 11 show a large reduction in scattering effects across the derivative spectrum after filtering. Extending the analysis to the *θ*_(±4 to ±5)_ optical configuration showed no ability to distinguish the lactose spectral feature before or after filtering.

## Discussion

Experimental results that were presented in the previous section systematically investigated the role of granular scattering in identification of materials in diffuse angles. In the forward-scattering geometry, *θ*_0_, the two-dimensional mapping of both *α*-lactose monohydrate and riboflavin was significantly improved after cepstrum filtering. However, the improvement was more pronounced at higher THz frequencies, where the spectral feature of riboflavin was completely obscured prior to the signal processing steps shown in Fig 2. When all diffuse angles, *θ*_(±1 to ±5)_, were considered, cepstrum analysis was able to eliminate the scattering artifacts and successfully resolve the absorption resonance of both lactose and riboflavin. Although the same cepstrum filters were used for the two materials in the entire analysis presented in this work, Figs 7–11, at higher scattering angles, the improvement in identification of riboflavin was markedly less than lactose. This effect was a result of two major factors. First, the granular scattering due to HDPE particles is proportional to approximately *f*^3^ in our study, and therefore, dramatically increases between 0.2 to 1 THz. Second, the absorption feature of riboflavin is broader and weaker than that of lactose. Consequently, as the signal to noise decreases with frequency, identification of riboflavin becomes much more challenging. Samples with larger granular sizes or roughness can redistribute energy to higher angles farther away from the forward-scattering direction.

As in all spectroscopic materials classification schemes, a user must have *a priori* knowledge of a target material’s spectral absorption feature. This is often the case for national security and standoff chemical detection applications for identifying illicit chemicals, such as drugs and explosives. The only prerequisites for implementing the cepstrum analysis technique described in this study is that a user must have the extinction coefficient of target materials and the THz spectra of the granular scattering media. This type of information can be found in literature [9, 38], THz spectral databases [39, 40], or through individual measurements. The cepstrum can be calculated from this information and a proper Gaussian filter can be designed to mitigate the effect of scattering. It should be noted, however, that the success of cepstrum filtering relies on the signal to noise and the relative spectral location of the absorption feature with respect to the level of frequency-dependent scattering.

## Conclusion

The study of electromagnetic scattering in the THz regime can enable the application of THz instruments in biophotonics, non-destructive testing, and standoff detection of chemicals. In this paper, we investigated the role of granular scattering in two-dimensional mapping of materials using diffuse scattered THz waves. We showed that appropriately chosen Gaussian filters in the quefrency domain can mitigate the frequency-dependent effects of granular scattering. The results indicate that the stronger resonance of lactose at lower THz frequencies was more successfully resolved compared to the weaker spectral feature of riboflavin at higher frequencies. Cepstrum analysis provides a signal processing technique to mitigate granular scattering when imaging in highly scattering media.
